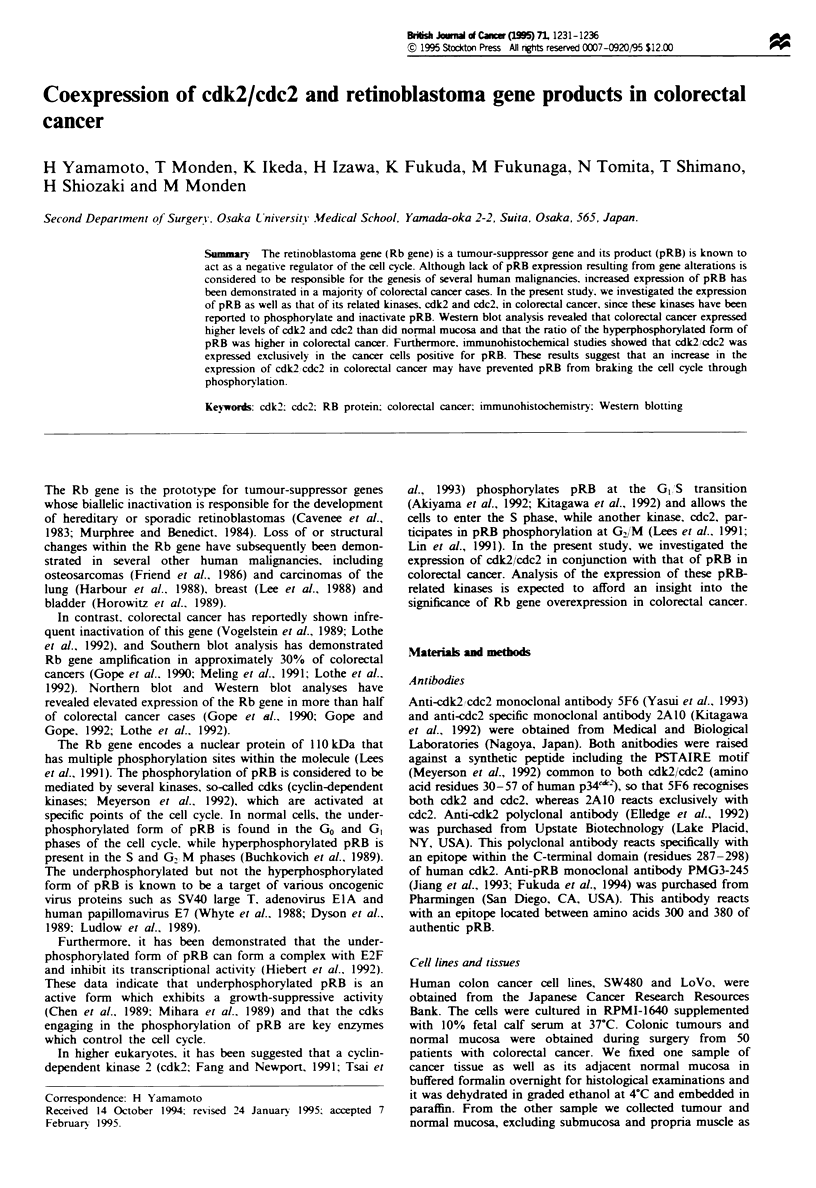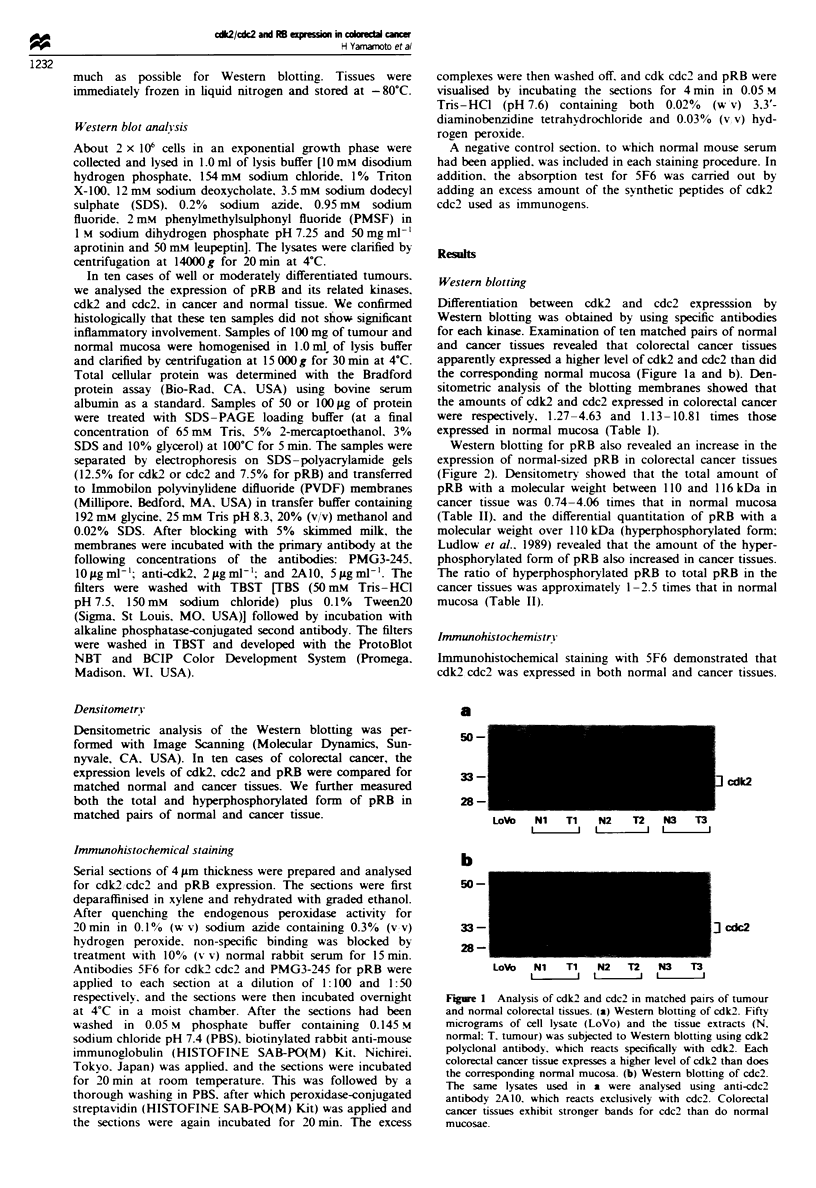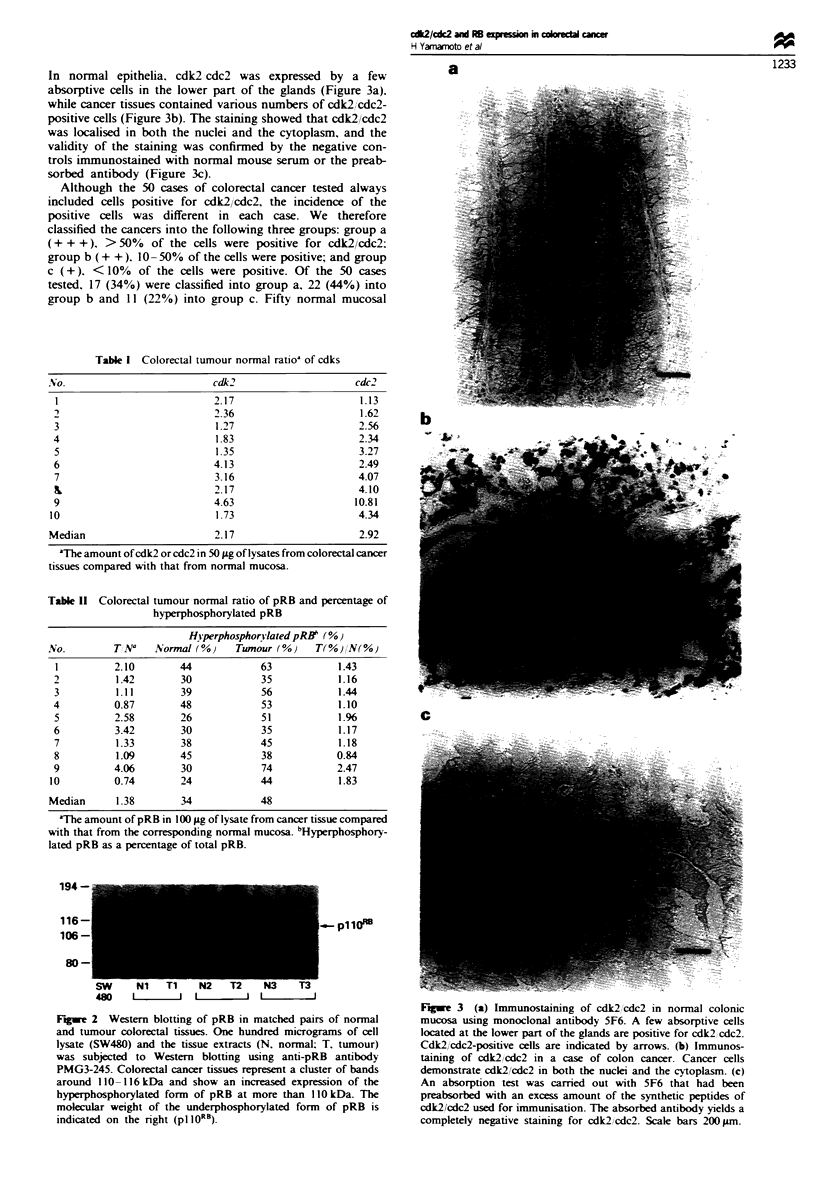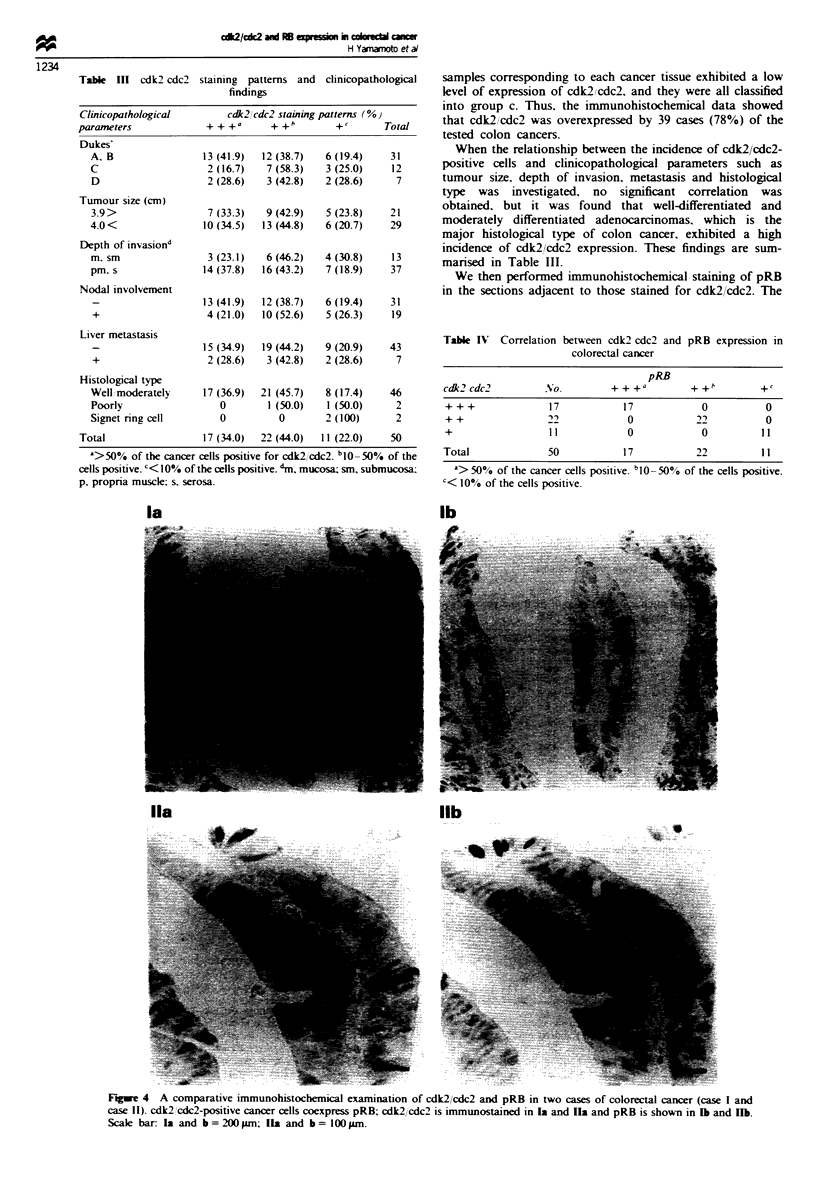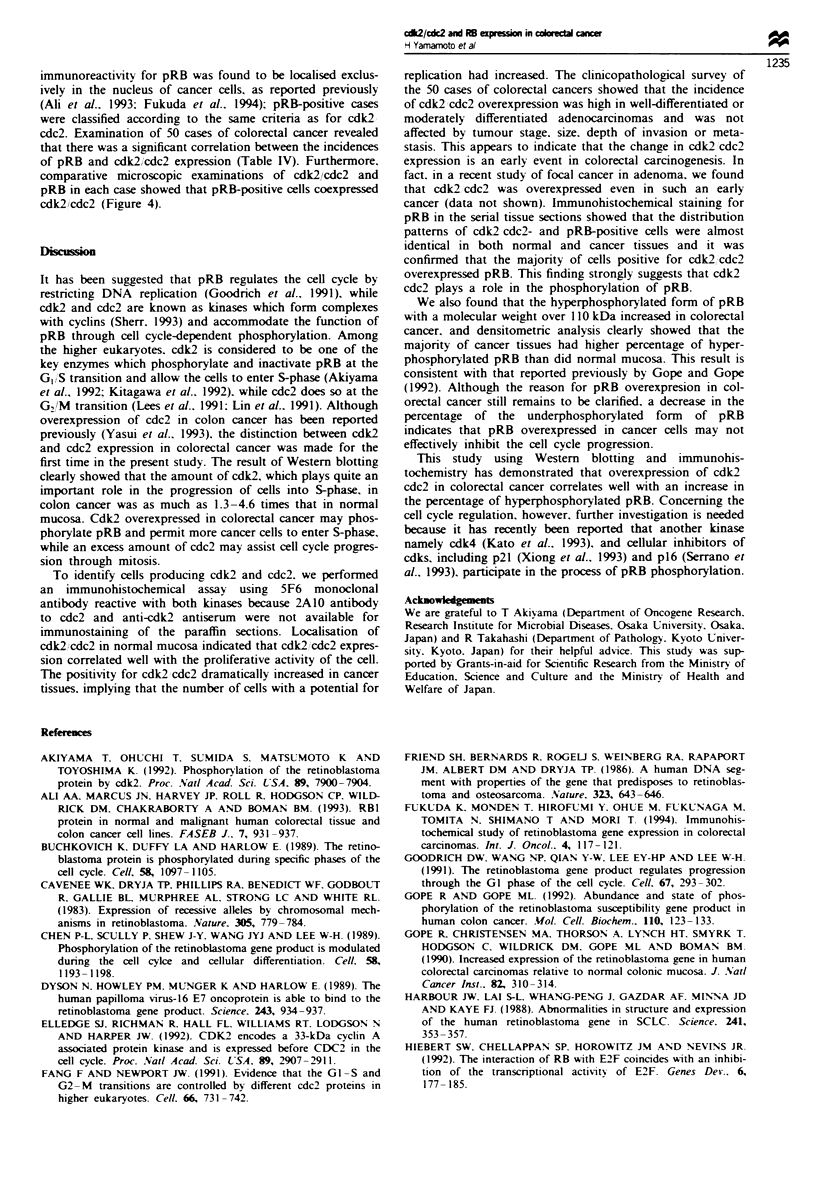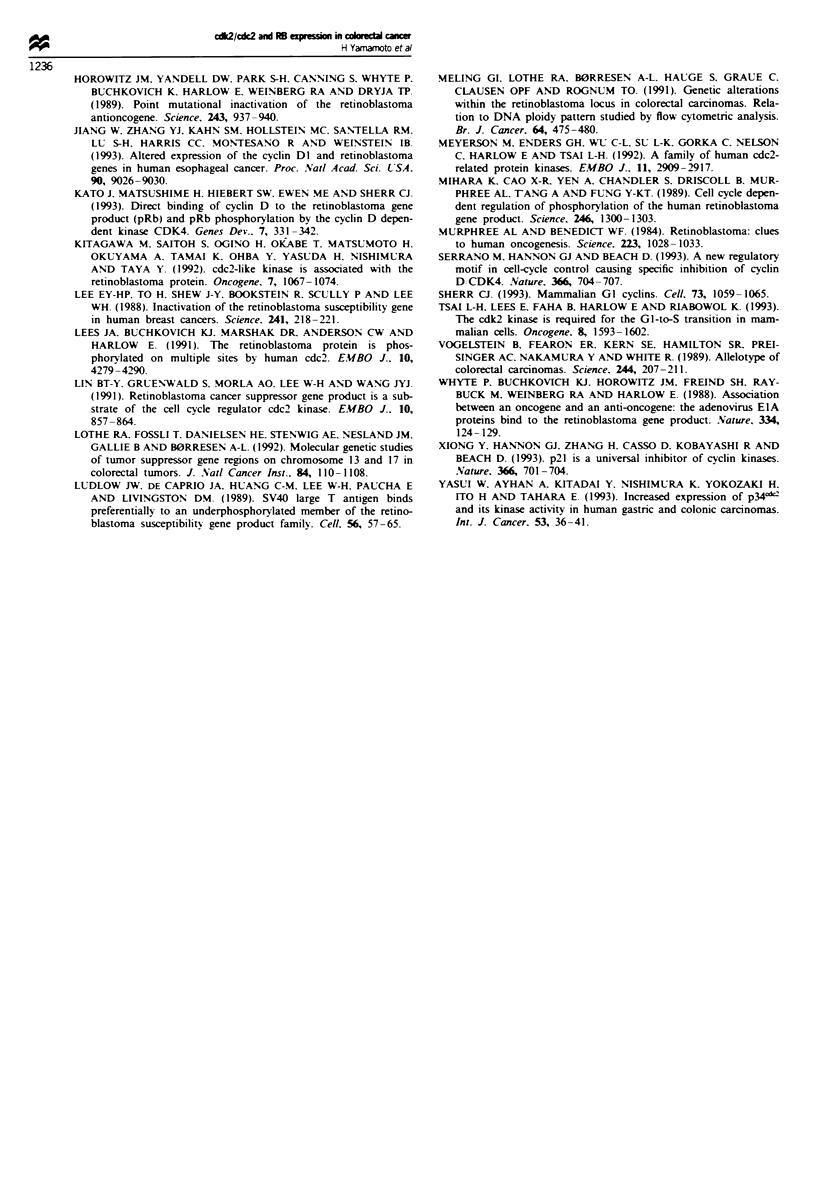# Coexpression of cdk2/cdc2 and retinoblastoma gene products in colorectal cancer.

**DOI:** 10.1038/bjc.1995.238

**Published:** 1995-06

**Authors:** H. Yamamoto, T. Monden, K. Ikeda, H. Izawa, K. Fukuda, M. Fukunaga, N. Tomita, T. Shimano, H. Shiozaki, M. Monden

**Affiliations:** Second Department of Surgery, Osaka University Medical School, Japan.

## Abstract

**Images:**


British Journal of Cancer (1995) 71, 1231-1236

© 1995 Stockton Press All rights reserved 0007-0920/95 $12.00

### Coexpression of cdk2/cdc2 and retinoblastoma gene products in colorectal cancer

H Yamamoto, T Monden, K Ikeda, H Izawa, K Fukuda, M Fukunaga, N Tomita, T Shimano, H Shiozaki and M Monden

*Second Department of Surgery, Osaka University Medical School, Yamada-oka 2-2, Suita, Osaka, 565, Japan.*

**Summary** The retinoblastoma gene (Rb gene) is a tumour-suppressor gene and its product (pRB) is known to act as a negative regulator of the cell cycle. Although lack of pRB expression resulting from gene alterations is considered to be responsible for the genesis of several human malignancies, increased expression of pRB has been demonstrated in a majority of colorectal cancer cases. In the present study, we investigated the expression of pRB as well as that of its related kinases, cdk2 and cdc2, in colorectal cancer, since these kinases have been reported to phosphorylate and inactivate pRB. Western blot analysis revealed that colorectal cancer expressed higher levels of cdk2 and cdc2 than did normal mucosa and that the ratio of the hyperphosphorylated form of pRB was higher in colorectal cancer. Furthermore, immunohistochemical studies showed that cdk2/cdc2 was expressed exclusively in the cancer cells positive for pRB. These results suggest that an increase in the expression of cdk2/cdc2 in colorectal cancer may have prevented pRB from braking the cell cycle through phosphorylation.

**Keywords**: cdk2; cdc2; RB protein; colorectal cancer; immunohistochemistry; Western blotting

The Rb gene is the prototype for tumour-suppressor genes whose biallelic inactivation is responsible for the development of hereditary or sporadic retinoblastomas (Cavenee *et al.*, 1983; Murphree and Benedict, 1984). Loss of or structural changes within the Rb gene have subsequently been demonstrated in several other human malignancies, including osteosarcomas (Friend *et al.*, 1986) and carcinomas of the lung (Harbour *et al.*, 1988), breast (Lee *et al.*, 1988) and bladder (Horowitz *et al.*, 1989).

In contrast, colorectal cancer has reportedly shown infrequent inactivation of this gene (Vogelstein *et al.*, 1989; Lothe *et al.*, 1992), and Southern blot analysis has demonstrated Rb gene amplification in approximately 30% of colorectal cancers (Gope *et al.*, 1990; Meling *et al.*, 1991; Lothe *et al.*, 1992). Northern blot and Western blot analyses have revealed elevated expression of the Rb gene in more than half of colorectal cancer cases (Gope *et al.*, 1990; Gope and Gope, 1992; Lothe *et al.*, 1992).

The Rb gene encodes a nuclear protein of 110 kDa that has multiple phosphorylation sites within the molecule (Lees *et al.*, 1991). The phosphorylation of pRB is considered to be mediated by several kinases, so-called cdks (cyclin-dependent kinases; Meyerson *et al.*, 1992), which are activated at specific points of the cell cycle. In normal cells, the underphosphorylated form of pRB is found in the $G_0$ and $G_1$ phases of the cell cycle, while hyperphosphorylated pRB is present in the S and $G_2/M$ phases (Buchkovich *et al.*, 1989). The underphosphorylated but not the hyperphosphorylated form of pRB is known to be a target of various oncogenic virus proteins such as SV40 large T, adenovirus E1A and human papillomavirus E7 (Whyte *et al.*, 1988; Dyson *et al.*, 1989; Ludlow *et al.*, 1989).

Furthermore, it has been demonstrated that the underphosphorylated form of pRB can form a complex with E2F and inhibit its transcriptional activity (Hiebert *et al.*, 1992). These data indicate that underphosphorylated pRB is an active form which exhibits a growth-suppressive activity (Chen *et al.*, 1989; Mihara *et al.*, 1989) and that the cdks engaging in the phosphorylation of pRB are key enzymes which control the cell cycle.

In higher eukaryotes, it has been suggested that a cyclin-dependent kinase 2 (cdk2; Fang and Newport, 1991; Tsai *et al.*, 1993) phosphorylates pRB at the $G_1/S$ transition (Akiyama *et al.*, 1992; Kitagawa *et al.*, 1992) and allows the cells to enter the S phase, while another kinase, cdc2, participates in pRB phosphorylation at $G_2/M$ (Lees *et al.*, 1991; Lin *et al.*, 1991). In the present study, we investigated the expression of cdk2/cdc2 in conjunction with that of pRB in colorectal cancer. Analysis of the expression of these pRB-related kinases is expected to afford an insight into the significance of Rb gene overexpression in colorectal cancer.

Correspondence: H Yamamoto
Received 14 October 1994; revised 24 January 1995; accepted 7 February 1995.

### Materials and methods

### *Antibodies*

Anti-cdk2/cdc2 monoclonal antibody 5F6 (Yasui *et al.*, 1993) and anti-cdc2 specific monoclonal antibody 2A10 (Kitagawa *et al.*, 1992) were obtained from Medical and Biological Laboratories (Nagoya, Japan). Both anitbodies were raised against a synthetic peptide including the PSTAIRE motif (Meyerson *et al.*, 1992) common to both cdk2/cdc2 (amino acid residues 30-57 of human $p34^{cdc2}$), so that 5F6 recognises both cdk2 and cdc2, whereas 2A10 reacts exclusively with cdc2. Anti-cdk2 polyclonal antibody (Elledge *et al.*, 1992) was purchased from Upstate Biotechnology (Lake Placid, NY, USA). This polyclonal antibody reacts specifically with an epitope within the C-terminal domain (residues 287-298) of human cdk2. Anti-pRB monoclonal antibody PMG3-245 (Jiang *et al.*, 1993; Fukuda *et al.*, 1994) was purchased from Pharmingen (San Diego, CA, USA). This antibody reacts with an epitope located between amino acids 300 and 380 of authentic pRB.

### *Cell lines and tissues*

Human colon cancer cell lines, SW480 and LoVo, were obtained from the Japanese Cancer Research Resources Bank. The cells were cultured in RPMI-1640 supplemented with 10% fetal calf serum at 37°C. Colonic tumours and normal mucosa were obtained during surgery from 50 patients with colorectal cancer. We fixed one sample of cancer tissue as well as its adjacent normal mucosa in buffered formalin overnight for histological examinations and it was dehydrated in graded ethanol at 4°C and embedded in paraffin. From the other sample we collected tumour and normal mucosa, excluding submucosa and propria muscle as much as possible for Western blotting. Tissues were immediately frozen in liquid nitrogen and stored at −80°C.

*Western blot analysis*

About $2 \times 10^6$ cells in an exponential growth phase were collected and lysed in 1.0 ml of lysis buffer [10 mM disodium hydrogen phosphate, 154 mM sodium chloride, 1% Triton X-100, 12 mM sodium deoxycholate, 3.5 mM sodium dodecyl sulphate (SDS), 0.2% sodium azide, 0.95 mM sodium fluoride, 2 mM phenylmethylsulphonyl fluoride (PMSF) in 1 M sodium dihydrogen phosphate pH 7.25 and 50 mg $ml^{-1}$ aprotinin and 50 mM leupeptin]. The lysates were clarified by centrifugation at 14000 *g* for 20 min at 4°C.

In ten cases of well or moderately differentiated tumours, we analysed the expression of pRB and its related kinases, cdk2 and cdc2, in cancer and normal tissue. We confirmed histologically that these ten samples did not show significant inflammatory involvement. Samples of 100 mg of tumour and normal mucosa were homogenised in 1.0 ml of lysis buffer and clarified by centrifugation at 15 000 *g* for 30 min at 4°C. Total cellular protein was determined with the Bradford protein assay (Bio-Rad, CA, USA) using bovine serum albumin as a standard. Samples of 50 or 100 μg of protein were treated with SDS–PAGE loading buffer (at a final concentration of 65 mM Tris, 5% 2-mercaptoethanol, 3% SDS and 10% glycerol) at 100°C for 5 min. The samples were separated by electrophoresis on SDS–polyacrylamide gels (12.5% for cdk2 or cdc2 and 7.5% for pRB) and transferred to Immobilon polyvinylidene difluoride (PVDF) membranes (Millipore, Bedford, MA, USA) in transfer buffer containing 192 mM glycine, 25 mM Tris pH 8.3, 20% (v/v) methanol and 0.02% SDS. After blocking with 5% skimmed milk, the membranes were incubated with the primary antibody at the following concentrations of the antibodies: PMG3-245, 10 μg $ml^{-1}$; anti-cdk2, 2 μg $ml^{-1}$; and 2A10, 5 μg $ml^{-1}$. The filters were washed with TBST [TBS (50 mM Tris–HCl pH 7.5, 150 mM sodium chloride) plus 0.1% Tween20 (Sigma, St Louis, MO, USA)] followed by incubation with alkaline phosphatase-conjugated second antibody. The filters were washed in TBST and developed with the ProtoBlot NBT and BCIP Color Development System (Promega, Madison, WI, USA).

*Densitometry*

Densitometric analysis of the Western blotting was performed with Image Scanning (Molecular Dynamics, Sunnyvale, CA, USA). In ten cases of colorectal cancer, the expression levels of cdk2, cdc2 and pRB were compared for matched normal and cancer tissues. We further measured both the total and hyperphosphorylated form of pRB in matched pairs of normal and cancer tissue.

*Immunohistochemical staining*

Serial sections of 4 μm thickness were prepared and analysed for cdk2/cdc2 and pRB expression. The sections were first deparaffinised in xylene and rehydrated with graded ethanol. After quenching the endogenous peroxidase activity for 20 min in 0.1% (w/v) sodium azide containing 0.3% (v/v) hydrogen peroxide, non-specific binding was blocked by treatment with 10% (v/v) normal rabbit serum for 15 min. Antibodies 5F6 for cdk2/cdc2 and PMG3-245 for pRB were applied to each section at a dilution of 1:100 and 1:50 respectively, and the sections were then incubated overnight at 4°C in a moist chamber. After the sections had been washed in 0.05 M phosphate buffer containing 0.145 M sodium chloride pH 7.4 (PBS), biotinylated rabbit anti-mouse immunoglobulin (HISTOFINE SAB-PO(M) Kit, Nichirei, Tokyo, Japan) was applied, and the sections were incubated for 20 min at room temperature. This was followed by a thorough washing in PBS, after which peroxidase-conjugated streptavidin (HISTOFINE SAB-PO(M) Kit) was applied and the sections were again incubated for 20 min. The excess complexes were then washed off, and cdk/cdc2 and pRB were visualised by incubating the sections for 4 min in 0.05 M Tris–HCl (pH 7.6) containing both 0.02% (w/v) 3,3′-diaminobenzidine tetrahydrochloride and 0.03% (v/v) hydrogen peroxide.

A negative control section, to which normal mouse serum had been applied, was included in each staining procedure. In addition, the absorption test for 5F6 was carried out by adding an excess amount of the synthetic peptides of cdk2/cdc2 used as immunogens.

### Results

*Western blotting*

Differentiation between cdk2 and cdc2 expresssion by Western blotting was obtained by using specific antibodies for each kinase. Examination of ten matched pairs of normal and cancer tissues revealed that colorectal cancer tissues apparently expressed a higher level of cdk2 and cdc2 than did the corresponding normal mucosa (Figure 1a and b). Densitometric analysis of the blotting membranes showed that the amounts of cdk2 and cdc2 expressed in colorectal cancer were respectively, 1.27–4.63 and 1.13–10.81 times those expressed in normal mucosa (Table I).

Western blotting for pRB also revealed an increase in the expression of normal-sized pRB in colorectal cancer tissues (Figure 2). Densitometry showed that the total amount of pRB with a molecular weight between 110 and 116 kDa in cancer tissue was 0.74–4.06 times that in normal mucosa (Table II), and the differential quantitation of pRB with a molecular weight over 110 kDa (hyperphosphorylated form; Ludlow *et al*., 1989) revealed that the amount of the hyperphosphorylated form of pRB also increased in cancer tissues. The ratio of hyperphosphorylated pRB to total pRB in the cancer tissues was approximately 1–2.5 times that in normal mucosa (Table II).

*Immunohistochemistry*

Immunohistochemical staining with 5F6 demonstrated that cdk2/cdc2 was expressed in both normal and cancer tissues.

**Figure 1** Analysis of cdk2 and cdc2 in matched pairs of tumour and normal colorectal tissues. (**a**) Western blotting of cdk2. Fifty micrograms of cell lysate (LoVo) and the tissue extracts (N, normal; T, tumour) was subjected to Western blotting using cdk2 polyclonal antibody, which reacts specifically with cdk2. Each colorectal cancer tissue expresses a higher level of cdk2 than does the corresponding normal mucosa. (**b**) Western blotting of cdc2. The same lysates used in **a** were analysed using anti-cdc2 antibody 2A10, which reacts exclusively with cdc2. Colorectal cancer tissues exhibit stronger bands for cdc2 than do normal mucosae.

In normal epithelia, cdk2/cdc2 was expressed by a few absorptive cells in the lower part of the glands (Figure 3a), while cancer tissues contained various numbers of cdk2/cdc2-positive cells (Figure 3b). The staining showed that cdk2/cdc2 was localised in both the nuclei and the cytoplasm, and the validity of the staining was confirmed by the negative controls immunostained with normal mouse serum or the preabsorbed antibody (Figure 3c).

Although the 50 cases of colorectal cancer tested always included cells positive for cdk2/cdc2, the incidence of the positive cells was different in each case. We therefore classified the cancers into the following three groups: group a (+ + +), >50% of the cells were positive for cdk2/cdc2; group b (+ +), 10–50% of the cells were positive; and group c (+), <10% of the cells were positive. Of the 50 cases tested, 17 (34%) were classified into group a, 22 (44%) into group b and 11 (22%) into group c. Fifty normal mucosal

**Table I** Colorectal tumour/normal ratio[a] of cdks

| *No.* | *cdk2* | *cdc2* |
|---|---|---|
| 1 | 2.17 | 1.13 |
| 2 | 2.36 | 1.62 |
| 3 | 1.27 | 2.56 |
| 4 | 1.83 | 2.34 |
| 5 | 1.35 | 3.27 |
| 6 | 4.13 | 2.49 |
| 7 | 3.16 | 4.07 |
| 8 | 2.17 | 4.10 |
| 9 | 4.63 | 10.81 |
| 10 | 1.73 | 4.34 |
| Median | 2.17 | 2.92 |

[a]The amount of cdk2 or cdc2 in 50 μg of lysates from colorectal cancer tissues compared with that from normal mucosa.

**Table II** Colorectal tumour/normal ratio of pRB and percentage of hyperphosphorylated pRB

| | | *Hyperphosphorylated pRB[b] (%)* | | |
|---|---|---|---|---|
| *No.* | *T/N[a]* | *Normal (%)* | *Tumour (%)* | *T(%)/N(%)* |
| 1 | 2.10 | 44 | 63 | 1.43 |
| 2 | 1.42 | 30 | 35 | 1.16 |
| 3 | 1.11 | 39 | 56 | 1.44 |
| 4 | 0.87 | 48 | 53 | 1.10 |
| 5 | 2.58 | 26 | 51 | 1.96 |
| 6 | 3.42 | 30 | 35 | 1.17 |
| 7 | 1.33 | 38 | 45 | 1.18 |
| 8 | 1.09 | 45 | 38 | 0.84 |
| 9 | 4.06 | 30 | 74 | 2.47 |
| 10 | 0.74 | 24 | 44 | 1.83 |
| Median | 1.38 | 34 | 48 | |

[a]The amount of pRB in 100 μg of lysate from cancer tissue compared with that from the corresponding normal mucosa. [b]Hyperphosphorylated pRB as a percentage of total pRB.

**Figure 2** Western blotting of pRB in matched pairs of normal and tumour colorectal tissues. One hundred micrograms of cell lysate (SW480) and the tissue extracts (N, normal; T, tumour) was subjected to Western blotting using anti-pRB antibody PMG3-245. Colorectal cancer tissues represent a cluster of bands around 110–116 kDa and show an increased expression of the hyperphosphorylated form of pRB at more than 110 kDa. The molecular weight of the underphosphorylated form of pRB is indicated on the right ($p110^{RB}$).

**Figure 3** (**a**) Immunostaining of cdk2/cdc2 in normal colonic mucosa using monoclonal antibody 5F6. A few absorptive cells located at the lower part of the glands are positive for cdk2/cdc2. Cdk2/cdc2-positive cells are indicated by arrows. (**b**) Immunostaining of cdk2/cdc2 in a case of colon cancer. Cancer cells demonstrate cdk2/cdc2 in both the nuclei and the cytoplasm. (**c**) An absorption test was carried out with 5F6 that had been preabsorbed with an excess amount of the synthetic peptides of cdk2/cdc2 used for immunisation. The absorbed antibody yields a completely negative staining for cdk2/cdc2. Scale bars 200 μm.

**Table III** cdk2/cdc2 staining patterns and clinicopathological findings

| *Clinicopathological parameters* | *cdk2/cdc2 staining patterns (%)* + + +[a] | + +[b] | +[c] | *Total* |
|---|---|---|---|---|
| Dukes' | | | | |
| A, B | 13 (41.9) | 12 (38.7) | 6 (19.4) | 31 |
| C | 2 (16.7) | 7 (58.3) | 3 (25.0) | 12 |
| D | 2 (28.6) | 3 (42.8) | 2 (28.6) | 7 |
| Tumour size (cm) | | | | |
| 3.9> | 7 (33.3) | 9 (42.9) | 5 (23.8) | 21 |
| 4.0< | 10 (34.5) | 13 (44.8) | 6 (20.7) | 29 |
| Depth of invasion[d] | | | | |
| m, sm | 3 (23.1) | 6 (46.2) | 4 (30.8) | 13 |
| pm, s | 14 (37.8) | 16 (43.2) | 7 (18.9) | 37 |
| Nodal involvement | | | | |
| − | 13 (41.9) | 12 (38.7) | 6 (19.4) | 31 |
| + | 4 (21.0) | 10 (52.6) | 5 (26.3) | 19 |
| Liver metastasis | | | | |
| − | 15 (34.9) | 19 (44.2) | 9 (20.9) | 43 |
| + | 2 (28.6) | 3 (42.8) | 2 (28.6) | 7 |
| Histological type | | | | |
| Well/moderately | 17 (36.9) | 21 (45.7) | 8 (17.4) | 46 |
| Poorly | 0 | 1 (50.0) | 1 (50.0) | 2 |
| Signet ring cell | 0 | 0 | 2 (100) | 2 |
| Total | 17 (34.0) | 22 (44.0) | 11 (22.0) | 50 |

[a]>50% of the cancer cells positive for cdk2/cdc2. [b]10–50% of the cells positive. [c]<10% of the cells positive. [d]m, mucosa; sm, submucosa; p, propria muscle; s, serosa.

samples corresponding to each cancer tissue exhibited a low level of expression of cdk2/cdc2, and they were all classified into group c. Thus, the immunohistochemical data showed that cdk2/cdc2 was overexpressed by 39 cases (78%) of the tested colon cancers.

When the relationship between the incidence of cdk2/cdc2-positive cells and clinicopathological parameters such as tumour size, depth of invasion, metastasis and histological type was investigated, no significant correlation was obtained, but it was found that well-differentiated and moderately differentiated adenocarcinomas, which is the major histological type of colon cancer, exhibited a high incidence of cdk2/cdc2 expression. These findings are summarised in Table III.

We then performed immunohistochemical staining of pRB in the sections adjacent to those stained for cdk2/cdc2. The

**Table IV** Correlation between cdk2/cdc2 and pRB expression in colorectal cancer

| *cdk2/cdc2* | *No.* | *pRB* + + +[a] | + +[b] | +[c] |
|---|---|---|---|---|
| + + + | 17 | 17 | 0 | 0 |
| + + | 22 | 0 | 22 | 0 |
| + | 11 | 0 | 0 | 11 |
| Total | 50 | 17 | 22 | 11 |

[a]> 50% of the cancer cells positive. [b]10–50% of the cells positive. [c]< 10% of the cells positive.

**Figure 4** A comparative immunohistochemical examination of cdk2/cdc2 and pRB in two cases of colorectal cancer (case I and case II). cdk2/cdc2-positive cancer cells coexpress pRB; cdk2/cdc2 is immunostained in **Ia** and **IIa** and pRB is shown in **Ib** and **IIb**. Scale bar: **Ia** and **b** = 200 μm; **IIa** and **b** = 100 μm.

immunoreactivity for pRB was found to be localised exclusively in the nucleus of cancer cells, as reported previously (Ali *et al.*, 1993; Fukuda *et al.*, 1994); pRB-positive cases were classified according to the same criteria as for cdk2/cdc2. Examination of 50 cases of colorectal cancer revealed that there was a significant correlation between the incidences of pRB and cdk2/cdc2 expression (Table IV). Furthermore, comparative microscopic examinations of cdk2/cdc2 and pRB in each case showed that pRB-positive cells coexpressed cdk2/cdc2 (Figure 4).

### Discussion

It has been suggested that pRB regulates the cell cycle by restricting DNA replication (Goodrich *et al.*, 1991), while cdk2 and cdc2 are known as kinases which form complexes with cyclins (Sherr, 1993) and accommodate the function of pRB through cell cycle-dependent phosphorylation. Among the higher eukaryotes, cdk2 is considered to be one of the key enzymes which phosphorylate and inactivate pRB at the $G_1/S$ transition and allow the cells to enter S-phase (Akiyama *et al.*, 1992; Kitagawa *et al.*, 1992), while cdc2 does so at the $G_2/M$ transition (Lees *et al.*, 1991; Lin *et al.*, 1991). Although overexpression of cdc2 in colon cancer has been reported previously (Yasui *et al.*, 1993), the distinction between cdk2 and cdc2 expression in colorectal cancer was made for the first time in the present study. The result of Western blotting clearly showed that the amount of cdk2, which plays quite an important role in the progression of cells into S-phase, in colon cancer was as much as 1.3–4.6 times that in normal mucosa. Cdk2 overexpressed in colorectal cancer may phosphorylate pRB and permit more cancer cells to enter S-phase, while an excess amount of cdc2 may assist cell cycle progression through mitosis.

To identify cells producing cdk2 and cdc2, we performed an immunohistochemical assay using 5F6 monoclonal antibody reactive with both kinases because 2A10 antibody to cdc2 and anti-cdk2 antiserum were not available for immunostaining of the paraffin sections. Localisation of cdk2/cdc2 in normal mucosa indicated that cdk2/cdc2 expression correlated well with the proliferative activity of the cell. The positivity for cdk2/cdc2 dramatically increased in cancer tissues, implying that the number of cells with a potential for replication had increased. The clinicopathological survey of the 50 cases of colorectal cancers showed that the incidence of cdk2/cdc2 overexpression was high in well-differentiated or moderately differentiated adenocarcinomas and was not affected by tumour stage, size, depth of invasion or metastasis. This appears to indicate that the change in cdk2/cdc2 expression is an early event in colorectal carcinogenesis. In fact, in a recent study of focal cancer in adenoma, we found that cdk2/cdc2 was overexpressed even in such an early cancer (data not shown). Immunohistochemical staining for pRB in the serial tissue sections showed that the distribution patterns of cdk2/cdc2- and pRB-positive cells were almost identical in both normal and cancer tissues and it was confirmed that the majority of cells positive for cdk2/cdc2 overexpressed pRB. This finding strongly suggests that cdk2/cdc2 plays a role in the phosphorylation of pRB.

We also found that the hyperphosphorylated form of pRB with a molecular weight over 110 kDa increased in colorectal cancer, and densitometric analysis clearly showed that the majority of cancer tissues had higher percentage of hyperphosphorylated pRB than did normal mucosa. This result is consistent with that reported previously by Gope and Gope (1992). Although the reason for pRB overexpresion in colorectal cancer still remains to be clarified, a decrease in the percentage of the underphosphorylated form of pRB indicates that pRB overexpressed in cancer cells may not effectively inhibit the cell cycle progression.

This study using Western blotting and immunohistochemistry has demonstrated that overexpression of cdk2/cdc2 in colorectal cancer correlates well with an increase in the percentage of hyperphosphorylated pRB. Concerning the cell cycle regulation, however, further investigation is needed because it has recently been reported that another kinase namely cdk4 (Kato *et al.*, 1993), and cellular inhibitors of cdks, including p21 (Xiong *et al.*, 1993) and p16 (Serrano *et al.*, 1993), participate in the process of pRB phosphorylation.

**Acknowledgements**
We are grateful to T Akiyama (Department of Oncogene Research, Research Institute for Microbial Diseases, Osaka University, Osaka, Japan) and R Takahashi (Department of Pathology, Kyoto University, Kyoto, Japan) for their helpful advice. This study was supported by Grants-in-aid for Scientific Research from the Ministry of Education, Science and Culture and the Ministry of Health and Welfare of Japan.

### References

AKIYAMA T, OHUCHI T, SUMIDA S, MATSUMOTO K AND TOYOSHIMA K. (1992). Phosphorylation of the retinoblastoma protein by cdk2. *Proc. Natl Acad. Sci. USA*, **89**, 7900–7904.

ALI AA, MARCUS JN, HARVEY JP, ROLL R, HODGSON CP, WILDRICK DM, CHAKRABORTY A AND BOMAN BM. (1993). RB1 protein in normal and malignant human colorectal tissue and colon cancer cell lines. *FASEB J.*, **7**, 931–937.

BUCHKOVICH K, DUFFY LA AND HARLOW E. (1989). The retinoblastoma protein is phosphorylated during specific phases of the cell cycle. *Cell*, **58**, 1097–1105.

CAVENEE WK, DRYJA TP, PHILLIPS RA, BENEDICT WF, GODBOUT R, GALLIE BL, MURPHREE AL, STRONG LC AND WHITE RL. (1983). Expression of recessive alleles by chromosomal mechanisms in retinoblastoma. *Nature*, **305**, 779–784.

CHEN P-L, SCULLY P, SHEW J-Y, WANG JYJ AND LEE W-H. (1989). Phosphorylation of the retinoblastoma gene product is modulated during the cell cylce and cellular differentiation. *Cell*, **58**, 1193–1198.

DYSON N, HOWLEY PM, MUNGER K AND HARLOW E. (1989). The human papilloma virus-16 E7 oncoprotein is able to bind to the retinoblastoma gene product. *Science*, **243**, 934–937.

ELLEDGE SJ, RICHMAN R, HALL FL, WILLIAMS RT, LODGSON N AND HARPER JW. (1992). CDK2 encodes a 33-kDa cyclin A associated protein kinase and is expressed before CDC2 in the cell cycle. *Proc. Natl Acad. Sci. USA*, **89**, 2907–2911.

FANG F AND NEWPORT JW. (1991). Evidence that the G1–S and G2–M transitions are controlled by different cdc2 proteins in higher eukaryotes. *Cell*, **66**, 731–742.

FRIEND SH, BERNARDS R, ROGELJ S, WEINBERG RA, RAPAPORT JM, ALBERT DM AND DRYJA TP. (1986). A human DNA segment with properties of the gene that predisposes to retinoblastoma and osteosarcoma. *Nature*, **323**, 643–646.

FUKUDA K, MONDEN T, HIROFUMI Y, OHUE M, FUKUNAGA M, TOMITA N, SHIMANO T AND MORI T. (1994). Immunohistochemical study of retinoblastoma gene expression in colorectal carcinomas. *Int. J. Oncol.*, **4**, 117–121.

GOODRICH DW, WANG NP, QIAN Y-W, LEE EY-HP AND LEE W-H. (1991). The retinoblastoma gene product regulates progression through the G1 phase of the cell cycle. *Cell*, **67**, 293–302.

GOPE R AND GOPE ML. (1992). Abundance and state of phosphorylation of the retinoblastoma susceptibility gene product in human colon cancer. *Mol. Cell. Biochem.*, **110**, 123–133.

GOPE R, CHRISTENSEN MA, THORSON A, LYNCH HT, SMYRK T, HODGSON C, WILDRICK DM, GOPE ML AND BOMAN BM. (1990). Increased expression of the retinoblastoma gene in human colorectal carcinomas relative to normal colonic mucosa. *J. Natl Cancer Inst.*, **82**, 310–314.

HARBOUR JW, LAI S-L, WHANG-PENG J, GAZDAR AF, MINNA JD AND KAYE FJ. (1988). Abnormalities in structure and expression of the human retinoblastoma gene in SCLC. *Science*, **241**, 353–357.

HIEBERT SW, CHELLAPPAN SP, HOROWITZ JM AND NEVINS JR. (1992). The interaction of RB with E2F coincides with an inhibition of the transcriptional activity of E2F. *Genes Dev.*, **6**, 177–185.

HOROWITZ JM, YANDELL DW, PARK S-H, CANNING S, WHYTE P, BUCHKOVICH K, HARLOW E, WEINBERG RA AND DRYJA TP. (1989). Point mutational inactivation of the retinoblastoma antioncogene. *Science*, **243**, 937-940.

JIANG W, ZHANG YJ, KAHN SM, HOLLSTEIN MC, SANTELLA RM, LU S-H, HARRIS CC, MONTESANO R AND WEINSTEIN IB. (1993). Altered expression of the cyclin D1 and retinoblastoma genes in human esophageal cancer. *Proc. Natl Acad. Sci. USA*, **90**, 9026-9030.

KATO J, MATSUSHIME H, HIEBERT SW, EWEN ME AND SHERR CJ. (1993). Direct binding of cyclin D to the retinoblastoma gene product (pRb) and pRb phosphorylation by the cyclin D dependent kinase CDK4. *Genes Dev.*, **7**, 331-342.

KITAGAWA M, SAITOH S, OGINO H, OKABE T, MATSUMOTO H, OKUYAMA A, TAMAI K, OHBA Y, YASUDA H, NISHIMURA AND TAYA Y. (1992). cdc2-like kinase is associated with the retinoblastoma protein. *Oncogene*, **7**, 1067-1074.

LEE EY-HP, TO H, SHEW J-Y, BOOKSTEIN R, SCULLY P AND LEE WH. (1988). Inactivation of the retinoblastoma susceptibility gene in human breast cancers. *Science*, **241**, 218-221.

LEES JA, BUCHKOVICH KJ, MARSHAK DR, ANDERSON CW AND HARLOW E. (1991). The retinoblastoma protein is phosphorylated on multiple sites by human cdc2. *EMBO J.*, **10**, 4279-4290.

LIN BT-Y, GRUENWALD S, MORLA AO, LEE W-H AND WANG JYJ. (1991). Retinoblastoma cancer suppressor gene product is a substrate of the cell cycle regulator cdc2 kinase. *EMBO J.*, **10**, 857-864.

LOTHE RA, FOSSLI T, DANIELSEN HE, STENWIG AE, NESLAND JM, GALLIE B AND BØRRESEN A-L. (1992). Molecular genetic studies of tumor suppressor gene regions on chromosome 13 and 17 in colorectal tumors. *J. Natl Cancer Inst.*, **84**, 110-1108.

LUDLOW JW, DE CAPRIO JA, HUANG C-M, LEE W-H, PAUCHA E AND LIVINGSTON DM. (1989). SV40 large T antigen binds preferentially to an underphosphorylated member of the retinoblastoma susceptibility gene product family. *Cell*, **56**, 57-65.

MELING GI, LOTHE RA, BØRRESEN A-L, HAUGE S, GRAUE C, CLAUSEN OPF AND ROGNUM TO. (1991). Genetic alterations within the retinoblastoma locus in colorectal carcinomas. Relation to DNA ploidy pattern studied by flow cytometric analysis. *Br. J. Cancer*, **64**, 475-480.

MEYERSON M, ENDERS GH, WU C-L, SU L-K, GORKA C, NELSON C, HARLOW E AND TSAI L-H. (1992). A family of human cdc2-related protein kinases. *EMBO J.*, **11**, 2909-2917.

MIHARA K, CAO X-R, YEN A, CHANDLER S, DRISCOLL B, MURPHREE AL, TANG A AND FUNG Y-KT. (1989). Cell cycle dependent regulation of phosphorylation of the human retinoblastoma gene product. *Science*, **246**, 1300-1303.

MURPHREE AL AND BENEDICT WF. (1984). Retinoblastoma: clues to human oncogenesis. *Science*, **223**, 1028-1033.

SERRANO M, HANNON GJ AND BEACH D. (1993). A new regulatory motif in cell-cycle control causing specific inhibition of cyclin D/CDK4. *Nature*, **366**, 704-707.

SHERR CJ. (1993). Mammalian G1 cyclins. *Cell*, **73**, 1059-1065.

TSAI L-H, LEES E, FAHA B, HARLOW E AND RIABOWOL K. (1993). The cdk2 kinase is required for the G1-to-S transition in mammalian cells. *Oncogene*, **8**, 1593-1602.

VOGELSTEIN B, FEARON ER, KERN SE, HAMILTON SR, PREISINGER AC, NAKAMURA Y AND WHITE R. (1989). Allelotype of colorectal carcinomas. *Science*, **244**, 207-211.

WHYTE P, BUCHKOVICH KJ, HOROWITZ JM, FREIND SH, RAYBUCK M, WEINBERG RA AND HARLOW E. (1988). Association between an oncogene and an anti-oncogene: the adenovirus E1A proteins bind to the retinoblastoma gene product. *Nature*, **334**, 124-129.

XIONG Y, HANNON GJ, ZHANG H, CASSO D, KOBAYASHI R AND BEACH D. (1993). p21 is a universal inhibitor of cyclin kinases. *Nature*, **366**, 701-704.

YASUI W, AYHAN A, KITADAI Y, NISHIMURA K, YOKOZAKI H, ITO H AND TAHARA E. (1993). Increased expression of $p34^{cdc2}$ and its kinase activity in human gastric and colonic carcinomas. *Int. J. Cancer*, **53**, 36-41.